# Contributions of lean mass and fat mass to bone mineral density: a study in postmenopausal women

**DOI:** 10.1186/1471-2474-11-59

**Published:** 2010-03-26

**Authors:** Lan T Ho-Pham, Nguyen D Nguyen, Thai Q Lai, Tuan V Nguyen

**Affiliations:** 1 Department of Internal Medicine, Pham Ngoc Thach University of Medicine, 86/2 Thanh Thai St, Ward 12, District 10, Ho Chi Minh City, Vietnam; 2 Department of Rheumatology, People's Hospital 115, 88 Thanh Thai Street, Ward 12, District 10, Ho Chi Minh City, Vietnam; 3 Osteoporosis and Bone Biology Program, Garvan Institute of Medical Research, 384 Victoria Street, Sydney NSW 2010, Australia; 4 St Vincent's Clinical School, Victoria Street, Sydney NSW 2010, Australia; 5 School of Public Health and Community Medicine, University of New South Wales, Sydney NSW 2052, Australia

## Abstract

**Background:**

The relative contribution of lean and fat to the determination of bone mineral density (BMD) in postmenopausal women is a contentious issue. The present study was undertaken to test the hypothesis that lean mass is a better determinant of BMD than fat mass.

**Methods:**

This cross-sectional study involved 210 postmenopausal women of Vietnamese background, aged between 50 and 85 years, who were randomly sampled from various districts in Ho Chi Minh City (Vietnam). Whole body scans, femoral neck, and lumbar spine BMD were measured by DXA (QDR 4500, Hologic Inc., Waltham, MA). Lean mass (LM) and fat mass (FM) were derived from the whole body scan. Furthermore, lean mass index (LMi) and fat mass index (FMi) were calculated as ratio of LM or FM to body height in metre squared (m^2^).

**Results:**

In multiple linear regression analysis, both LM and FM were independent and significant predictors of BMD at the spine and femoral neck. Age, lean mass and fat mass collectively explained 33% variance of lumbar spine and 38% variance of femoral neck BMD. Replacing LM and FM by LMi and LMi did not alter the result. In both analyses, the influence of LM or LMi was greater than FM and FMi. Simulation analysis suggested that a study with 1000 individuals has a 78% chance of finding the significant effects of both LM and FM, and a 22% chance of finding LM alone significant, and zero chance of finding the effect of fat mass alone.

**Conclusions:**

These data suggest that both lean mass and fat mass are important determinants of BMD. For a given body size -- measured either by lean mass or height --women with greater fat mass have greater BMD.

## Background

Several prospective studies in the last three decades have consistently indicated that bone mineral density (BMD) is the best indicator of fracture risk [1,2]. Each standard deviation decrease in BMD is associated with an approximately 2-fold increase in fracture risk [3], and the increase is more pronounced in hip fracture [1] than in non-hip fracture. Therefore, the diagnosis of postmenopausal osteoporosis is largely based on a measurement of BMD [4]. Furthermore, BMD is highly related to body weight, such that individuals with higher body weight have higher BMD [5] and reduced fracture risk [6]. Body weight explains approximately 30% of variance in BMD, making it one of the best determinants of BMD [7,8].

Body weight is largely made up of two components: fat mass (FM) and lean mass (LM, or fat-free mass). The relative contribution of these two components to the variation in BMD has been highly contentious. While some studies have suggested that LM, not FM, is associated with BMD [9-18]; other studies have shown that FM, not LM, is an important determinant of BMD [19-22]. Still other studies have found that both fat mass and lean mass were significant predictors of bone density [23-25], with lean mass being more important predictor than fat mass in pre-menopausal women, and fat mass a more important than lean mass in post-menopausal women [24]. The inconsistency of findings may relate to the expression of bone mass as an areal BMD or apparent BMD, and the collinearity between FM and LM [9].

Most previous studies were based on Caucasian populations, and the results can not necessarily be extrapolated to Asian populations, whose body fat is believed to be higher than in Caucasian populations [26]. We asked two specific questions: (i) is lean mass more important that fat mass as a determinant of bone mineral density in postmenopausal women; and (ii) given the sampling variability in the correlation between lean mass and fat mass, what is the chance of detecting the effect of either lean mass or fat mass on bone mineral density. This study was designed to address the two research questions.

## Methods

### Study setting and participants

The study setting was Ho Chi Minh city (formerly Saigon), a major city and an economic hub in Vietnam. The study was designed as a cross-sectional investigation, in which 210 women aged between 50 and 85 were randomly sampled by the cluster sampling scheme. None of the participants had any diseases deemed to affect osteoporosis (such as hyperthyroidism, hyperparathyroidism, renal failure, malabsorption syndrome, alcoholism, chronic colitis, multiple myeloma, leukemia, chronic arthritis) or previous use of therapies that interfere with bone metabolism (e.g., glucocorticoids, heparin, warfarin, thyroxine, estrogen). The study protocol and procedures were approved by the ethics committee of Hospital 115 and Pham Ngoc Thach University of Medicine.

### Bone mineral density measurement

BMD was measured at the lumbar spine (LS), femoral neck (FN) and whole body (WB) in all participants. The measurement was done with a dual energy X-ray absorptiometry (DXA) densitometer (QDR 4500, Hologic Inc., Waltham, MA). The precision error (%CV) in our laboratory was 2% for lumbar spine and 1.8% for femoral neck BMD, and 1.5% for whole body BMD. Fat tissue mass (FM) and lean tissue mass (LM) were derived from the whole body scan. In addition, we calculated the percent body fat (PBF) by dividing FM by body weight. The densitometer was standardized by a standard phantom every time before measurement.

Anthropometrical parameters including age, weight, and standing height were obtained. Body weight was measured by using an electronic balance with indoor clothing without shoes. Height was determined without shoes on a portable stadiometer with mandible plane parallel to the floor.

### Data analysis

We were specifically interested in the following question: for a given body size, what is the association between fat mass and BMD? Therefore, the primary purpose of analysis was to assess the association between lean mass, fat mass, and BMD. However, since body size is associated with all of these measures, any association between lean mass or fat mass and BMD should ideally be adjusted for body size. In this study, we chose height, rather than weight, as a proxy for body size, because the correlation between fat mass and height (r = 0.25) is lower than the correlation between fat mass and weight (r = 0.80). We derived the fat mass index (FMi) and lean mass index (LMi) by the following formulae: FMi = FM/(height)^a ^and LMi = LM/(height)^b^, where height is expressed in meters [27]. The power constants *a *and *b *were derived by fitting the equation of log FM or log LM against height: log(FM) = *k *+ *a*×log(height), and log(LM) = *c *+ *b*×log(height). Using the observed data from our study, we found *a = *1.96 and *b *= 1.70, which is close to 2. Thus, FMi = FM/(height)^2 ^and LMi = LM/(height)^2 ^was calculated, which is similar to the calculation of body mass index. Multiple linear regression analysis was used to analyze the relative contributions of FMi and LMi to BMD.

In the next analysis, we addressed the following question: if the present study were repeated many times, what would be the distribution or variability of the effects of lean mass and fat mass on BMD, and how would sample size affect the distribution? In order to address the question, we conducted a simulation study, in which 10,000 pseudo studies were generated, with each study having a sample size of 50, 100, 200, 300, 400, 500, and 1000 women. In each study, weight, height, lean mass, fat mass, and lumbar spine BMD were generated using the means, variance-covariance matrix of the real study. In each study, lumbar spine BMD was modeled as a linear function of lean mass and fat mass in the multiple linear regression, from which the statistical significance (*P *< 0.05) of each regression parameters was noted. The distribution of regression parameters and their statistical significance were then obtained. All analyses and simulation were done with the R program [28]. The R codes used for the simulation study are available from the first author.

## Results

The study included 210 participants, whose anthropometric and demographic characteristics are shown in Table 1. The average age of participants was 62 years, with range being 50 to 85. All women were postmenopausal, with years since menopause being 14.3 (range: 1 to 41). The average age of menopause was 48 years. The average fat mass in the entire sample was 18.8 kg, which is 35% of body weight. There was a significant correlation between lean mass and fat mass (*r *= 0.37; *P *< 0.0001). Advancing age was associated with decreased lean mass (*r *= -0.18; *P *= 0.008), but with a non-significantly increased fat mass (*r *= 0.06; *P *= 0.39). As a result, there was a significant positive correlation between age and percent body fat (*r *= 0.15; *P *= 0.02).

**Table 1:** Anthropometric and densitometric characteristics of participants (n = 210)

Variable	Mean (SD)	Range
Age (year)	62.0 (9.56)	50 - 85
Years since menopause (years)	14.3 (10.0)	1 - 41
Weight (kg)	53.3 (7.9)	33 - 75
Height (cm)	148.9 (5.7)	132 - 165
Body mass index (kg/m^2^)	24.1 (3.2)	15 - 34
Lean mass (kg)	32.3 (4.1)	23.1 - 53.0
Fat mass (kg)	18.8 (4.9)	5.2 - 34.1
Percent body fat (%)	35.0 (6.18)	15.8 - 66.2
Lumbar spine BMD (g/cm^2^)	0.76 (0.14)	0.41 - 1.17
Femoral neck BMD (g/cm^2^)	0.63 (0.11)	0.38 - 1.06
Whole body BMD (g/cm^2^)	0.89 (0.11)	0.58 - 1.22

### Lean mass, fat mass, and bone density

**Figure 1:**
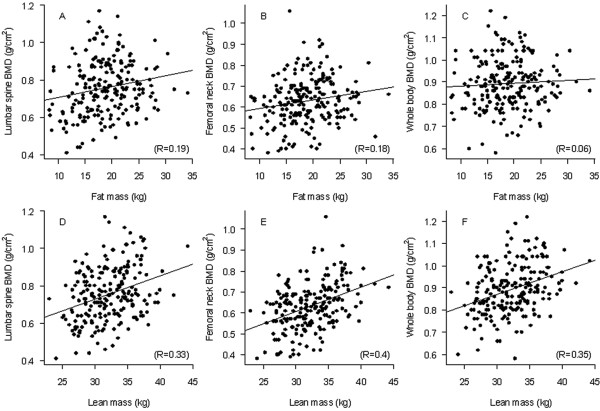
Correlation between fat mass and BMD at the (A) lumbar spine, (B) femoral neck, (C) whole body, and between lean mass and BMD at the (D) lumbar spine, (E) femoral neck, and (F) whole body.

In univariate analysis, greater lean mass or fat mass was associated with greater BMD at the lumbar spine, femoral neck, and whole body (Figure 1). In the multiple linear regression analysis, lean mass remained the strongest predictor of BMD at all sites (Table 2). Independent of age, each 5 kg increase in lean mass was associated with 0.034, 0.031, and 0.036 g/cm^2 ^increase in BMD at the lumbar spine, femoral neck, and whole body, respectively. However, each 5 kg increase in fat mass was associated with a 0.022, 0.017, and 0.001 g/cm^2 ^increase in BMD at the lumbar spine, femoral neck, and whole body, respectively. In fact, after accounting for lean mass, the association of fat mass and whole body BMD was not statistically significant (*P *= 0.90). Age, lean mass, and fat mass collectively "explained" 33% variance in lumbar spine BMD and 38% variance in femoral neck BMD. When lean mass and fat mass were replaced by lean mass index and fat mass index (Model 4), similar associations were also observed. However, a simpler model with only weight or BMI could also explain the same amount of variance in BMD.

**Table 2:** Determinants of bone mineral density: results of multiple linear regression analysis

Model and determinant	Bone mineral density (g/cm^2^)		
	
	Lumbar spine	Femoral neck	Whole body
**Model 1: Age and body weight**			
Age (5 years)	**-0.036 (0.004)**	**-0.029 (0.003)**	**-0.032 (0.003)**
Weight (5 kg)	**0.027 (0.005)**	**0.022 (0.004)**	**0.016 (0.004)**
R-squared	0.33	0.38	0.37

**Model 2: Age and height**			
Age (5 years)	**-0.032 (0.005)**	**-0.028 (0.003)**	**-0.029 (0.003)**
Height (5 cm)	**0.027 (0.008)**	**0.012 (0.006)**	**0.016 (0.006)**
R-squared	0.29	0.30	0.34

**Model 3: Age, body weight, height**			
Age (5 years)	**-0.034 (0.004)**	**-0.030 (0.003)**	**-0.031 (0.003)**
Weight (5 kg)	**0.023 (0.006)**	**0.024 (0.004)**	**0.015 (0.004)**
Height (5 cm)	0.012 (0.008)	-0.005 (0.006)	0.006 (0.006)
R-squared	0.34	0.38	0.37

**Model 4: Age and BMI**			
Age	**-0.039 (0.004)**	**-0.032 (0.003)**	**-0.034 (0.003)**
BMI	**0.031 (0.008)**	**0.032 (0.058)**	**0.019 (0.006)**
R-squared	0.30	0.37	0.35

**Model 5: Age, lean mass, fat mass**			
Age (5 years)	**-0.036 (0.004)**	**-0.029 (0.003)**	**-0.030 (0.003)**
Lean mass (5 kg)	**0.034 (0.011)**	**0.031 (0.008)**	**0.036 (0.008)**
Fat mass (5 kg)	**0.022 (0.009)**	**0.017 (0.007)**	0.001 (0.006)
R-squared	0.33	0.38	0.39

Model 6: Age, lean mass index, fat mass index*			
Age (5 years)	**-0.039 (0.004)**	**-0.033 (0.003)**	**-0.033 (0.003)**
Lean mass index (kg/m^2^)	**0.010 (0.006)**	**0.014 (0.004)**	**0.015 (0.004)**
Fat mass index (kg/m^2^)	**0.009 (0.004)**	**0.008 (0.003)**	0.000 (0.003)
R-squared	0.29	0.36	0.36

Notes: All bolded figures indicate that the association was statistically significant at p < 0.05 level. (1) lean mass index = lean mass/height^2^, and fat mass index = fat mass/height^2 ^(see Methods section).

**Figure 2:**
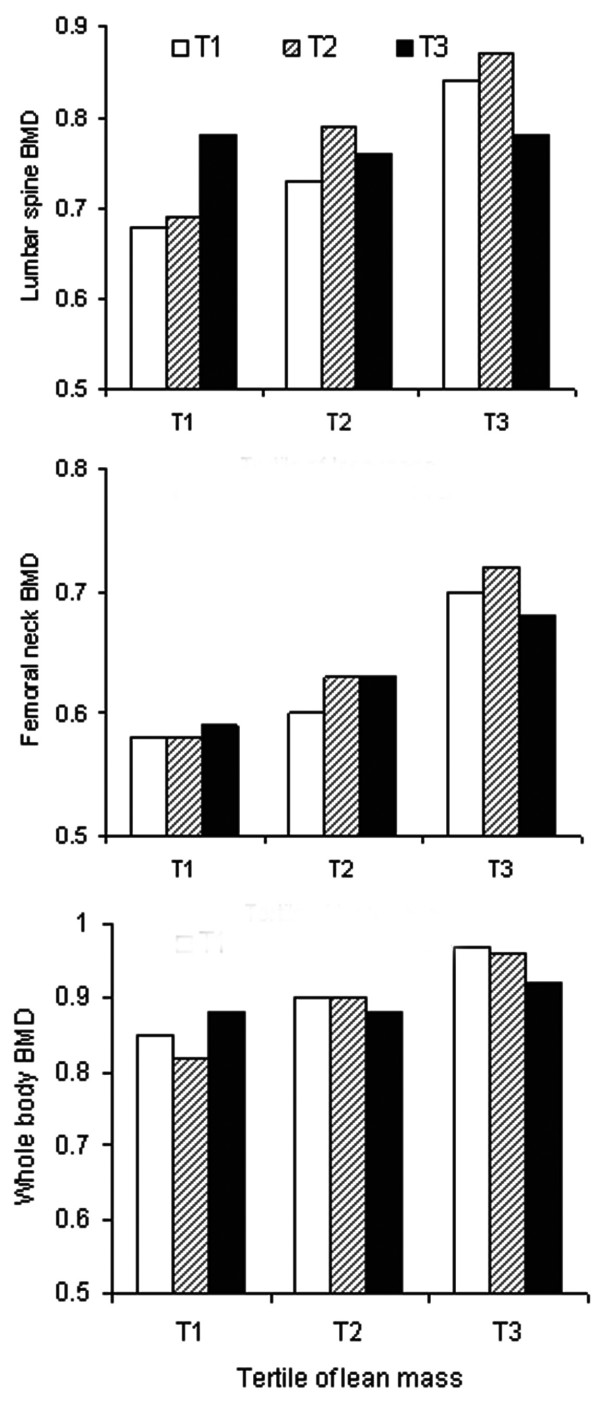
**Mean lumbar spine (top panel), femoral neck (middle panel), and whole body BMD (bottom panel) for a tertile lean mass (x-axis) and tertile fat mass**. The tertiles of lean mass were as follows: < 30.4 kg, 30.5 to 34 kg, and > 34.1 kg. Tertiles of fat mass were as follows: < 16.7 kg, 16.8 to 21.2 kg, and > 21.3 kg. The standard deviation for each bar (subgroup) for lumbar spine BMD was ~0.14 g/cm^2^, and for femoral neck and whole body was ~0.11 g/cm^2^.

In a further analysis, we estimated mean and standard deviation of BMD stratified by tertile of lean mass and fat mass. Results of this analysis (Figure 2) confirm the results of regression analysis: lean mass had a greater influence on BMD than fat mass. For example, individuals in the third tertile of lean mass (34 - 53 kg) had on average 0.09 g/cm^2 ^higher lumbar spine BMD than those in the first tertile of lean mass (23 - 30 kg); but the difference in BMD between the first tertile of fat mass (5 - 17 kg) and the third tertile of fat mass (21 - 34 kg) was 0.05 g/cm^2^. Moreover, for a given fat mass, individuals with more lean mass had greater BMD at all sites.

### Simulation analysis

**Figure 3:**
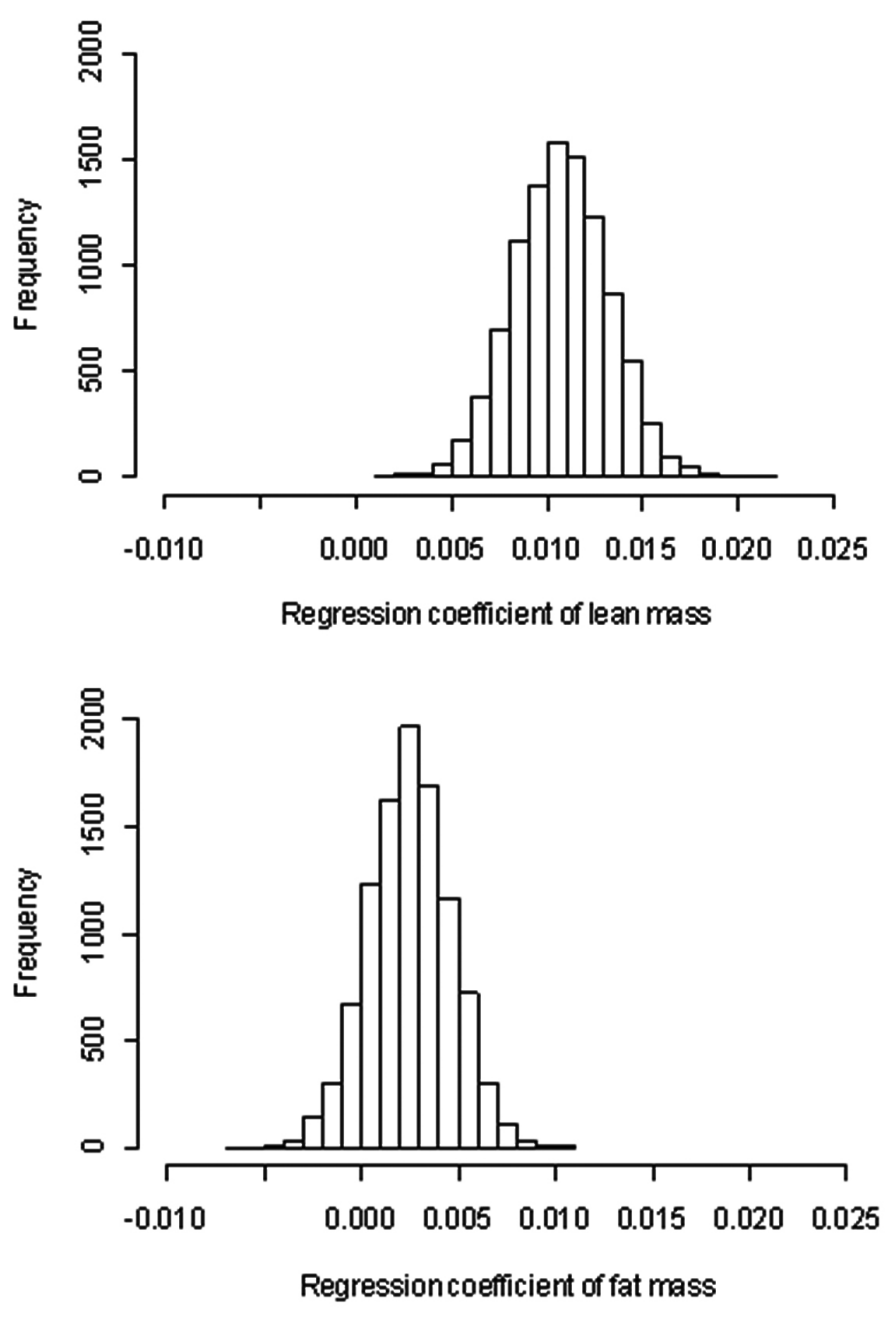
**Distribution of regression coefficients associated with lean mass (top panel) and fat mass (bottom panel)**. The regression model was LSBMD = *a *+ *b*(lean mass) + *c*(fat mass), and the figure presents the distribution of possible values of *b *and *c *in 10000 pseudo studies, with each study having 210 individuals.

The distribution of 10,000 simulated regression coefficients associated with LM and FM is shown in Figure 3. The figure shows that each 1 kg increase in lean mass was associated with a ~0.03 g/cm^2 ^increase in lumbar spine BMD, which was about three times the increase associated with a 1 kg of fat mass. The distribution of regression coefficients associated with fat mass suggested that there is a 10% chance that the coefficient of fat mass could be negative. However, virtually 100% regression coefficients associated with lean mass were positive.

Results of further simulation analyses are shown in Table 3. As expected, as the sample size increases, the width of confidence interval of all correlation coefficients becomes narrower. The simulation analysis suggested that lean mass is consistently a better predictor of bone density than fat mass. Further analyses suggest that a study with 50 individuals has a 53% chance of finding a significant effect of lean mass without an effect of fat mass, 6.3% chance of finding a significant effect of fat mass without an effect of lean mass, and 3% chance of showing significant effects of both lean and fat mass. The probability of finding significant effects of both lean mass and fat mass progressively increases with larger sample size. On the other hand, as the sample size increases, the chance to detect a significant of fat mass alone declines. For example, a study with 1000 individuals has a 78% chance of detecting the effect of both lean mass and fat mass, and a 22% chance of detecting the effect of lean mass without an effect of fat mass. A 'wrong" model with weight and percent body fat (PBF) as predictors could yield a negative regression of PBF and BMD.

**Table 3:** Results of simulation studies: distribution of correlations between variables, regression coefficients, and frequency of "significance" results

Parameters	Sample size						
	
	50	100	200	300	400	500	1000
**Correlation between**							
Fat mass and weight*	0.83	0.83	0.83	0.83	0.83	0.83	0.83
	0.74	0.77	0.79	0.80	0.80	0.80	0.81
	0.89	0.88	0.86	0.86	0.85	0.85	0.84
Lean mass and weight*	0.80	0.80	0.80	0.80	0.80	0.80	0.80
	0.70	0.73	0.76	0.76	0.77	0.77	0.78
	0.87	0.85	0.84	0.83	0.83	0.82	0.82
Fat mass and lean mass*	0.37	0.37	0.37	0.37	0.37	0.37	0.37
	0.15	0.22	0.27	0.28	0.29	0.30	0.32
	0.56	0.50	0.46	0.45	0.43	0.43	0.41
Fat mass and LSBMD*	0.21	0.20	0.20	0.20	0.20	0.20	0.20
	-0.04	0.04	0.09	0.11	0.12	0.13	0.15
	0.42	0.35	0.31	0.29	0.28	0.27	0.25
Lean mass and LSBMD*	0.34	0.34	0.34	0.34	0.34	0.34	0.34
	0.12	0.19	0.24	0.26	0.27	0.27	0.29
	0.53	0.48	0.44	0.42	0.41	0.40	0.38

Regression coefficient**							
Lean mass	0.011	0.011	0.011	0.011	0.011	0.011	0.011
	0.002	0.005	0.007	0.007	0.008	0.008	0.009
	0.019	0.015	0.015	0.014	0.014	0.013	0.013
Fat mass	0.003	0.002	0.003	0.003	0.003	0.003	0.003
	-0.005	-0.002	-0.001	-0.000	0.000	0.000	0.001
	0.010	0.007	0.006	0.005	0.005	0.005	0.004

**Frequency (%) of "significance"**^†^							
Not lean mass, not fat mass	37.4	10.9	0.3	0.02	0.0	0.0	0.0
Lean mass, not fat mass	52.9	75.6	75.6	67.7	58.8	50.6	22.1
Fat mass, not lean mass	6.3	3.8	0.5	0.05	0.0	0.0	0.0
Lean mass and fat mass	3.4	9.7	23.6	32.2	41.2	49.4	77.8

*For each pair of variables, the values are average coefficient (first row), lower 95% confidence interval (second row) and upper 95% confidence interval (third row).

Ten thousand pseudo-studies were simulated; each study had 50, 100, 200, 300, 400, 500, and 1000 individuals. The results presented here represent summaries of 10,000 iterations.

**The model considered was: LSBMD = *a *+ *b*(lean mass) + *c*(fat mass). The figures shown here are estimates of *b *(for lean mass) and *c *(for fat mass). For each parameter, the numbers are median, 5th and 95th percentiles (in brackets).

^†^The "significance" was defined as P < 0.05 for each or both regression coefficients.

## Discussion

The relative contribution of lean mass and fat mass to bone mineral density remains a contentious issue. While a majority of studies have found a positive association between lean mass and BMD [9-18], few studies have shown that FM is an important determinant of BMD [19-22]. By using an appropriate adjustment for body size, we have shown in this study that both lean and fat mass were significantly associated with BMD, with the former being a stronger predictor than the latter. We have also shown by simulation that with adequate sample size, the probability of finding significant effects of both lean mass and fat mass is as high as 78%. These results deserve further comments.

Distinguishing the role of lean mass versus fat mass as a determinant of BMD has clinical relevance, because an association between BMD and LM suggests that increase in physical activity may directly translate into protection against osteoporosis; while an association between BMD and FM implies that obesity may have protective effect against bone loss. However, delineating the independent effects of LM and FM on BMD is not straightforward, because the two measurements are correlated, and depending on the magnitude of correlation, when they are considered in a multiple linear regression model, it is sometimes impossible to separate one effect from another.

It was initially reported that fat mass was a major determinant of BMD [19], but subsequent studies could not confirm that association [9-18]. However, in that initial study, the correlation between lean mass and BMD (*r *= 0.55) was not much different from the correlation between fat mass and BMD (*r *= 0.60), and when the two variables were statistically significant in the multiple linear regression. Our results suggest that both lean mass and fat mass are positively associated with BMD, even though the correlation between the two body composition measures was moderate (*r *= 0.37), which is consistent with previous studies [19]. However, our results further suggest that the influence of lean mass on BMD is consistently greater than that of fat mass. Indeed, variation in lean mass 'explained" approximately 13% variance in femoral neck BMD, whereas variation in fat mass explained only 4% variance in femoral neck BMD. Assuming that lean mass is a proxy measure of muscle mass, the present result implies that muscle mass, and by extrapolation, changes in physical activity may have a greater effect on bone mass than changes in fat mass does.

Statistically, whether lean mass or fat mass is associated with BMD is dependent on the inter-correlations among lean mass, fat mass, and BMD. However, the correlation could be different among studies due to sample size and sampling variability. In such a heuristic situation, simulation is a reasonable approach to gain insight into the variability. Using the observed correlations among the three variables, we have conducted a simulation study, and found that studies with 200 individuals or less have between 1 and 6% chance of finding a significant effect of fat mass without a significant effect of lean mass, and 55% to 68% chance of finding the effect of lean mass without a significant effect of fat mass. However, studies with more than 200 individuals have a much better chance (32% to 78%) of detecting the effects of both lean mass and fat mass. These results are in broad agreement with the literature so far, in which the effects of both lean mass and fat mass were reported in studies with at least 300 individuals, and only small studies reported the effect of fat mass alone on BMD. Our finding has important implications for the design of future studies, in which sample size is a crucial factor for the identification and delineation of the effects of lean mass and fat mass on BMD.

It has recently been suggested that for a given body weight, there was a negative correlation between fat mass and BMD [12,29]. This suggestion was based on the BMD predicted from a least squares regression model with weight and percent body fat being predictors. However, it has been pointed out that the use of weight as an adjustment for the effect of fat mass on BMD is not appropriate and could yield fallacious result, because fat mass is a component of, and is therefore highly correlated with, body weight [27]. Moreover, fat mass is a component of body weight, when both fat mass and weight are considered in a regression model, it will give misleading results due to the problem of mathematical coupling [30,31] which has been raised since the early 20th century [32]. It has been shown that height (not weight) is an appropriate proxy of body size. Therefore, the effect of fat mass or lean mass on BMD should be adjusted for body height. In this study, after adjusting for body height, we found that both lean mass and fat mass had independent effects on BMD. We have further shown that for a given lean mass, individuals with greater fat mass had greater BMD, suggesting that fat mass is indeed independently and positively associated with BMD.

Body mass index is often used as a predictor or a factor of adjusting BMD in the literature. However, we found that this usage is sub-optimal, because of lack of empirical evidence and difficulty in interpretation. Empirically, we found that weight and height are better determinants of BMD than BMI is. For example, in our study, body weight and height collectively explained ~15% of the variance of lumbar spine and femoral neck BMD, whereas BMI explained only 3 to 7% of the BMD variance. Since BMI is derived as the ratio of weight over height squared, a positive association between BMI and BMD in a regression model without height implies that within levels of weight, height is inversely associated with BMD in a quadratic fashion. For example, model 2 (Table 2) implies that for a given weight, BMD is linearly related to 1/height^2 ^with regression coefficient of 0.034 (for lumbar spine BMD). Such an inverse relation of height to BMD is hardly biologically interpretable. In other words, a model with BMI does not capture adequately the relationship between BMD and body size.

The present results should be interpreted within a number of potential strengths and weaknesses. The participants were randomly drawn from the general population which should ensure its external validity. The DXA measurements of fat mass, lean mass and bone mass are accurate and reliable, which ensure the internal validity of the study. However, the women were of Vietnamese background, among whom lifestyles, nutritional and physical activity may differ from other populations. The study design was cross-sectional, and it is not possible to make any cause-and-effect inference on the relationship between lean mass, fat mass and BMD.

## Conclusions

These data suggest that both lean mass and fat mass are important determinants of BMD, with the former having a greater influence on BMD than the latter. For a given body size (measured by either lean mass or height), women with greater fat mass have greater BMD. Because lean mass is related to physical activity, this finding re-inforces the concept that physical activity is an important component in the prevention of bone loss and osteoporosis in postmenopausal women.

## Abbreviations

All abbreviations are defined in the text.

## Competing interests

All authors declare that they have no competing interests with regard to this work. Professor T. Nguyen received honorarium for speaking and providing consultant services to MSD Vietnam Ltd, Sanofi-Aventis, Norvatis, and Roche.

## Authors' contributions

Contributions of the authors to the manuscript included *Study concept and design: *Lan Ho-Pham and Tuan Nguyen; *Acquisition of data: *Lan Ho-Pham and Thai Q. Lai. *Analysis and interpretation of data: *Lan Ho-Pham, Nguyen Nguyen, Thai Q. Lai, and Tuan Nguyen; *Drafting the manuscript: *Lan Ho-Pham, Tuan Nguyen and Nguyen Nguyen; *Statistical analysis: *Nguyen Nguyen, Thai Q. Lai and Tuan Nguyen; *Critical revision of the manuscript: *Lan Ho-Pham, Tuan Nguyen and Nguyen Nguyen, Thai Q. Lai. All authors read and approved the final manuscript.

## Pre-publication history

The pre-publication history for this paper can be accessed here:

http://www.biomedcentral.com/1471-2474/11/59/prepub
